# Biochemical analysis of TssK, a core component of the bacterial Type VI secretion system, reveals distinct oligomeric states of TssK and identifies a TssK–TssFG subcomplex

**DOI:** 10.1042/BJ20131426

**Published:** 2014-06-26

**Authors:** Grant English, Olwyn Byron, Francesca R. Cianfanelli, Alan R. Prescott, Sarah J. Coulthurst

**Affiliations:** *Division of Molecular Microbiology, College of Life Sciences, University of Dundee, Dow Street, Dundee DD1 5EH, U.K.; ‡Division of Cell Signalling and Immunology, College of Life Sciences, University of Dundee, Dow Street, Dundee DD1 5EH, U.K.; †College of Medical, Veterinary and Life Sciences, University of Glasgow, Sir Graeme Davies Building, 120 University Place, Glasgow G12 8QQ, U.K.

**Keywords:** bacterial protein secretion, native complex isolation, protein oligomerization, *Serratia marcescens*, Type VI secretion system (T6SS), AAA+, ATPase associated with diverse cellular activities, AUC, analytical ultracentrifugation, DDM, dodecyl maltoside, EAEC, enteroaggregative *Escherichia coli*, IMAC, immobilized ion-affinity chromatography, MBP, maltose-binding protein, RNAPβ, RNA polymerase β, SE, sedimentation equilibrium, SEC, size-exclusion chromatography, SV, sedimentation velocity, TEV, tobacco etch virus, T6SS, Type VI secretion system

## Abstract

Gram-negative bacteria use the Type VI secretion system (T6SS) to inject toxic proteins into rival bacteria or eukaryotic cells. However, the mechanism of the T6SS is incompletely understood. In the present study, we investigated a conserved component of the T6SS, TssK, using the antibacterial T6SS of *Serratia marcescens* as a model system. TssK was confirmed to be essential for effector secretion by the T6SS. The native protein, although not an integral membrane protein, appeared to localize to the inner membrane, consistent with its presence within a membrane-anchored assembly. Recombinant TssK purified from *S. marcescens* was found to exist in several stable oligomeric forms, namely trimer, hexamer and higher-order species. Native-level purification of TssK identified TssF and TssG as interacting proteins. TssF and TssG, conserved T6SS components of unknown function, were required for T6SS activity, but not for correct localization of TssK. A complex containing TssK, TssF and TssG was subsequently purified *in vitro*, confirming that these three proteins form a new subcomplex within the T6SS. Our findings provide new insight into the T6SS assembly, allowing us to propose a model whereby TssK recruits TssFG into the membrane-associated T6SS complex and different oligomeric states of TssK may contribute to the dynamic mechanism of the system.

## Abstract 2

Genetic and biochemical analysis of TssK, an essential component of the bacterial Type VI secretion system, revealed that it forms a new subcomplex with two other core components, TssF and TssG, and displays several oligomerization states *in vitro*.

## INTRODUCTION

Protein secretion by Gram-negative bacteria is the controlled transport of selected proteins from the cytoplasm, across the cell envelope and out of the cell. The secreted proteins may be released into the extracellular environment or injected directly into target cells. Protein secretion is performed by six classes of specialized proteinaceous machinery, the Type I–Type VI secretion systems (T1SS–T6SS) [1]. Secreted proteins enable bacteria to interact closely with their surroundings, with eukaryotic host organisms and with other bacteria, and they play a central role in bacterial virulence [2]. T6SSs are the most recently described of the Gram-negative bacterial secretion systems and are widely distributed among diverse species. The T6SS has been shown to play an important role in pathogenicity towards eukaryotic host cells in a variety of important human pathogens, including *Pseudomonas aeruginosa*, *Burkholderia mallei*, *Vibrio cholerae* and *Aeromonas hydrophila* [3–8]. Most notably, the action of several of these anti-eukaryotic systems appears to result in disruption of the actin cytoskeleton [8,9]. It is now clear that the T6SS can be also used to target other, competitor, bacteria and is thus likely to play an important role in polymicrobial infections. Antibacterial activity has been reported for T6SSs of multiple bacterial species, including *P. aeruginosa*, *V. cholerae* and *Burkholderia thailandensis* [10–12]. Various Type VI-secreted antibacterial toxins have recently been identified. The biggest group are peptidoglycan hydrolase enzymes which attack the cell wall of target bacteria, including several families of peptidoglycan amidase and glycoside hydrolase enzymes [13,14]. Additionally, a superfamily of phospholipase effectors, attacking the target cell membrane, were described recently and a small number of other, unrelated, effector toxins have been experimentally identified, but are yet to be fully characterized [13,15–17]. We reported previously that the opportunistic pathogen *Serratia marcescens* Db10 possesses a single T6SS with potent strain-specific antibacterial activity [18]. We have shown recently that this antibacterial activity is provided, at least in part, by six T6SS-dependent secreted effector proteins, Ssp1–Ssp6, all toxins inhibitory to other bacterial strains [15,19]. *S. marcescens* avoids self-killing by the production of cognate immunity proteins which provide specific protection against the toxicity of the different effectors. For example, Ssp1 and Ssp2 are peptidoglycan amidase enzymes whose activity is specifically inhibited by high-affinity binding of the immunity proteins Rap1a and Rap2a respectively, forming heterotetrameric complexes in which the effector active site is blocked by the immunity protein [19,20]. Since *S. marcescens* possesses a single constitutively active T6SS, it provides a convenient system to study the basic mechanism of this important secretion system.

T6SSs contain 13 conserved components (TssA–TssM) which are thought to form the core apparatus and to enable effector proteins to be injected into target cells in a single cell-contact-dependent step [21–23]. Various biochemical and structural data support a model in which T6SS components form several subassemblies that interact to form a dynamic inverted bacteriophage-like injection machine. The first is a membrane-associated complex that transverses the periplasm and anchors the T6SS in the cell envelope. This subassembly is composed of two integral membrane proteins, TssL and TssM, and an outer membrane periplasmic-facing lipoprotein, TssJ, where TssM interacts with TssL and TssJ, through its large periplasmic C-terminal domain, to form a membrane-spanning channel [24–27]. The cytoplasmic domain of TssM has been shown to display ATPase activity and is thought to play an energizing role in the system [28]. The T6SS is anchored to the cell wall by TssL either through its own periplasmic peptidoglycan-binding domain or through its interaction with the TagL accessory protein [29,30]. The second subassembly is a bacteriophage-like structure containing the extracellular components Hcp (TssD) and VgrG (TssI), which are structurally related to bacteriophage T4 tube and tailspike proteins respectively. Hcp proteins form hexameric ring-shaped structures that can polymerize and assemble as tube-like structures, and a trimer of VgrG is believed to cap the top of the Hcp tube and puncture target cell membranes [31–34]. It has been proposed recently that small PAAR (Pro-Ala-Ala-Arg) domain proteins may form the final sharp tip of the needle [35]. This ‘needle’ is thought to be surrounded by a tubular complex composed of TssB (VipA) and TssC (VipB), forming an intracellular contractile phage sheath-like structure [36–38]. Extended and contracted conformations of this tubule structure have been observed by microscopy, and contraction is thought to eject the Hcp–VgrG structure out of the secreting cell and into a target cell, thereby allowing the translocation of effector molecules [36]. Additionally, this tubular structure was observed to be connected to the inner membrane by a cytoplasmic bell-shaped ‘basal’ structure that is yet to be characterized [36]. Contracted TssBC tubular complexes are recognized and disassembled by the AAA+ (ATPase associated with diverse cellular activities) protein TssH (ClpV) that has also been shown to remodel these complexes *in vitro* in the presence of ATP. This remodelling activity should allow recycling of the apparatus for new rounds of secretion and also reverse non-productive sheath formation in the cytoplasm [38,39].

Considering the five remaining core T6SS components, TssE has detectable similarity with the phage gp25 baseplate protein [40], but no function has been readily assigned to the other proteins (TssA, TssF, TssG and TssK). It is thought that these five proteins may be located in the cytoplasm and form part of the basal structure that can be observed by microscopy. The TssK protein has been shown previously to be essential for T6SS activity in systematic mutagenesis studies in *Edwardsiella tarda*, *V. cholerae* and *Agrobacterium tumefaciens* [41,42]. In addition, a recent study has confirmed that TssK is required for T6SS activity in EAEC (enteroaggregative *Escherichia coli*) and suggested that it interacts with several components of the membrane complex and phage-like subassemblies [43].

The aim of the present study was to investigate the biochemical properties, cellular localization and interaction partners of the TssK protein of the *S. marcescens* T6SS. We aimed to study TssK, as far as possible, at native levels and in the native organism, in particular to obtain physiologically relevant localization and interaction data. We demonstrate that TssK is essential for T6SS activity and exhibits several stable oligomeric states. Additionally, analysis of the native protein is used to show that TssK localizes with the inner membrane, apparently as part of a stable T6SS assembly, and to identify TssF and TssG as interaction partners of TssK. Finally, a stable complex containing TssF, TssG and TssK can be isolated *in vitro* and may represent a new subcomplex within the basal structure of the T6SS.

## EXPERIMENTAL

### Bacterial strains, culture conditions and plasmids

Bacterial strains and plasmids used in the present study are detailed in Table 1. *S. marcescens* was routinely grown with good aeration in LB medium (10 g/l tryptone, 5 g/l yeast extract and 5 g/l NaCl) at 30°C. When required, media were supplemented with antibiotics: ampicillin (100 μg/ml), kanamycin (100 μg/ml) and chloramphenicol (25 μg/ml). *S. marcescens* chromosomal mutants with in-frame deletions in selected genes or introducing a chromosomal His_6_ tag fused at the N-terminus or C-terminus of *tssK* (*SMA2253*) were constructed by marker (allelic) exchange using the suicide vector pKNG101 [44] as described previously [18]. Plasmids for complementing the expression of the deleted gene *in trans* were derived from pSUPROM or pSC100. For protein overexpression and purification, *tssK* with a C-terminal TEV (tobacco etch virus) protease-cleavable His_6_ tag was cloned in pSC102. For co-purification of the TssKFG complex, *tssF* with an N-terminal TEV protease-cleavable His_6_ tag and untagged *tssG* were cloned in site 1 of pACYCDuet-1 (NcoI/BamHI, BamHI/EcoRI), and untagged *tssK* was cloned in site 2 (NdeI/XhoI). Molecular biological techniques were performed according to standard protocols [45] and manufacturers’ instructions. Oligonucleotide primers were obtained from Sigma–Genosys, molecular biology reagents were purchased from leading suppliers (Roche, NEB, Invitrogen and Sigma) and synthetic DNA sequences used in the construction of pSC168 were generated by Dundee Cell Products.

**Table 1:** Bacterial strains and plasmids used in the present study Ap, ampicillin; Cm, chloramphenicol; Kn, kanamycin; Sm, streptomycin.

Strain or plasmid	Genotype or relevant features	Source or reference
Strains		
*Serratia marcescens* Db10	Wild-type strain	Laboratory stock
SJC3	Db10 Δ*clpV* (ΔSMA2274, TssH)	[18]
SJC10	Db10 Δ*lip* (ΔSMA2252, TssJ)	[18]
SJC11	Db10 Δ*tssE* (ΔSMA2271, TssE)	[18]
MJF1	Db10 *tssK*–His_6_ (SMA2253 with a C-terminal His_6_ tag)	The present study
SJC23	Db10 His_6_–*tssK* (SMA2253 with an N-terminal His_6_ tag)	The present study
GE04	Db10 Δ*tssK* (ΔSMA2253)	The present study
GE05	MJF1 Δ*tssG* (ΔSMA2273)	The present study
GE06	MJF1 Δ*tssF* (ΔSMA2272)	The present study
Plasmids		
pSUPROM	Plasmid for constitutive expression of cloned genes under the control of the *E. coli tat* promoter, Kn^R^	[56]
pACYCDuet-1	Protein coexpression plasmid containing two cloning sites, each preceded by an IPTG-inducible T7 promoter, Cm^R^	Novagen
pSC045	*tssE* (SMA2271) coding sequence in pSUPROM, Kn^R^	[18]
pSC100	Medium-copy protein-overexpression plasmid derived from pQE80, IPTG-inducible T5 promoter, permits fusion of His_6_ tag to the N- or C-terminus of the overexpressed protein, Ap^R^, Kn^R^	The present study
pSC102	Medium-copy protein-overexpression plasmid derived from pQE80, IPTG-inducible T5 promoter, permits fusion of a His_6_ tag followed by a TEV protease cleavage site to the N- or C-terminus of the overexpressed protein, Ap^R^, Kn^R^	The present study
pSC119	*tssK* (SMA2253) coding sequence in pSC102, generating a fusion protein with a TEV-cleavable C-terminal His_6_ tag, Ap^R^, Kn^R^	The present study
pSC132	Marker exchange plasmid for generation of chromosomal *tssK*–His_6_ fusion, in pKNG101, Sm^R^	The present study
pSC167	*tssG* (SMA2273) and *tssK* (SMA2253) coding sequences in pACYCDuet-1 (in sites 1 and 2 respectively), directing expression of untagged TssG and TssK (precursor of pSC168)	The present study
pSC168	*tssF* (SMA2272), *tssG* (SMA2273) and *tssK* (SMA2253) coding sequences in pACYCDuet-1 (*tssF* and *G* in site 1, *tssK* in site 2), directing expression of TssG and TssK without any tags and TssF fused to an N-terminal His_6_ tag followed by a TEV protease cleavage site	The present study
pSC521	*tssK* (SMA2253) coding sequence in pSC100, generating a fusion protein with a C-terminal His_6_ tag, Ap^R^, Kn^R^	The present study
pSC522	*tssK* (SMA2253) coding in pSC100, generating a fusion protein with an N-terminal His_6_ tag, Ap^R^, Kn^R^	The present study
pSC532	Marker exchange plasmid for generation of in-frame deletion of *tssK*, in pKNG101, Sm^R^	The present study
pSC554	Marker exchange plasmid for generation of in-frame deletion of *tssF*, in pKNG101, Sm^R^	The present study
pSC555	Marker exchange plasmid for generation of in-frame deletion of *tssG*, in pKNG101, Sm^R^	The present study
pKNG101	Suicide plasmid for marker exchange, *sacBR*, mobRK2, oriR6K, Sm^R^	[44]
pBluescript KS+	High-copy-number cloning plasmid, multiple cloning site in *lacZ*', Ap^R^	Stratagene

### Immunodetection of proteins

For detection of secreted proteins, anti-Hcp immunoblots were performed as described in [18] and anti-Ssp2 immunoblots were carried out as described in [19]. Anti-TssK rabbit polyclonal antibody was raised against the purified protein (Dundee Cell Products) and used at 1:1000 dilution, and anti-TssJ antibody [27] was used at 1:4000 dilution, both using horseradish-peroxidase-conjugated goat anti-rabbit secondary antibody (Thermo Fisher Scientific) at 1:10000 dilution. Anti-RNAPβ (RNA polymerase β) antibody (Neoclone) was used at 1:20000 dilution, and anti-MBP (maltose-binding protein) antibody (NEB) was used at 1:10000 dilution; both with horseradish-peroxidase-conjugated goat anti-mouse secondary antibody (Roche) at 1:10000 dilution.

### Fractionation to determine native localization of TssK

Bacterial strains were grown at 30°C for 5 h. The cells from 5 ml of culture were incubated with 0.5 ml of 0.5 M Tris/HCl (pH 7.8) for 10 min at room temperature, then harvested by centrifugation at 2773 ***g*** for 10 min at 4°C. Cell pellets were washed once with 5 ml of LB buffer then resuspended in 1 ml of ice-cold osmotic shock buffer [30 mM Tris/HCl, pH 7.8, 40% (w/v) sucrose and 2 mM EDTA]. This represented the whole-cell fraction. After 10 min of incubation at 30°C, pellets were collected by centrifugation at 14100 ***g*** for 10 min at 4°C, resuspended quickly with 900 μl of ice-cold MilliQ water and kept on ice for 10 min. The supernatant collected after centrifugation at 14100 ***g*** for 10 min at 4°C contained the periplasmic fraction and the pellet was resuspended in 900 μl of 50 mM Tris/HCl (pH 7.8) for sonication. Samples were lysed on ice by sonication for five 15 s bursts (25% amplitude) at 30 s intervals, and the unbroken cells and cellular debris were removed by centrifugation at 14100 ***g*** for 5 min at 4°C. The supernatant was recovered and represented the crude cell extract. The extract (750 μl) was treated with urea or carbonate to release proteins loosely or non-specifically associated with the membranes, followed by an ultracentrifugation step at 200000 ***g*** for 30 min at 4°C. The supernatant was recovered and represented the soluble cytoplasmic fraction. The remaining pellet, containing inner and outer membranes, was resuspended in 750 μl of 50 mM Tris/HCl (pH 7.8) and represented the total membrane fraction. A 100 μl sample of each cellular fraction was mixed with 100 μl of 2× gel sample buffer [18] and analysed by SDS/PAGE (15% gel) and immunoblotting. Equivalent amounts, on an individual cell basis, of each fraction in each strain were assayed. Custom anti-TssK polyclonal antibody was used to detect TssK, anti-RNAPβ antibody was used as the cytoplasmic marker, anti-MBP antibody was used as the periplasmic marker and anti-TssJ antibody was used as the membrane marker, as above.

To examine the nature of membrane association, total membranes (prepared without prior treatment of the crude cell extract) were incubated with 50 mM Tris/HCl (pH 7.8) containing 6 M urea, 1 M NaCl or 0.2 M Na_2_CO_3_ for 1 h at room temperature and were then subjected to another ultracentrifugation step. Alternatively, membranes were solubilized with 50 mM Tris/HCl (pH 7.8) containing 2% (w/v) DDM (dodecyl maltoside), 2% (w/v) CHAPS or 2% (v/v) Triton X-100 detergents and, following incubation for 2 h at 4°C, solubilized membrane proteins were separated from insoluble proteins by ultracentrifugation at 200000 ***g*** for 30 min at 4°C.

Inner and outer bacterial membranes were separated using discontinuous sedimentation sucrose gradients based on the method described in [24,46]. Wild-type *S. marcescens* total membranes were prepared from 500 ml of culture and resuspended in 0.5 ml of 20% (w/v) sucrose containing a 1:200 dilution of EDTA-free protease inhibitor cocktail set III (Calbiochem). The membrane fraction was then loaded on to the top of a discontinuous sucrose gradient composed of nine 1.2 ml layers of sucrose (from top to bottom), i.e. 30%, 35%, 37.5%, 40%, 42.5%, 45%, 50%, 55% and 70%, and subjected to ultracentrifugation at 100000 ***g*** for 70 h at 4°C in a SW 41 Ti rotor (Beckman). Fractions of 0.5 ml were collected from the top and every second fraction was analysed by SDS/PAGE (15% gel) and immunoblotting using anti-TssK and anti-TssJ antibodies.

### Protein overexpression and purification

Recombinant TssK protein was produced in *S. marcescens* Db10 freshly transformed with plasmid pSC119. Transformant colonies were used to inoculate 5 ml of LB medium with kanamycin and grown overnight at 30°C. From this, 2.5 ml was used to inoculate 500 ml of LB medium supplemented with kanamycin and IPTG at a final concentration of 1 mM in 2.5 ml baffled flasks and grown at 30°C with good aeration for 8 h. Cells were harvested by centrifugation at 4000 ***g*** for 45 min at 4°C, washed with 20 ml of 50 mM Tris/HCl (pH 7.5) and stored at −80°C. The thawed cell pellet was resuspended in lysis buffer (50 mM Tris/HCl, pH 7.5, containing a 1:200 dilution of EDTA-free protease inhibitor cocktail set III; 4 ml/g of cell pellet) and incubated at room temperature for 15 min. The cells were transferred to a glass tissue homogenizer and ground slowly on ice to avoid foam formation. The cells were then lysed with four passes through an Emulsiflex French press-style high pressure cell disruptor [20000 psi (1 psi=6.9 kPa)] and cell debris was removed by centrifugation at 14100 ***g*** for 30 min at 4°C. TssK–His_6_ was initially isolated by IMAC (immobilized metal-ion-affinity chromatography). The supernatant (soluble protein fraction) was loaded on to a 5 ml HisTrap™ HP chelating column (GE Healthcare) pre-loaded with Ni^2+^ at a flow rate of 1 ml/min using the ÄKTApurifier™ FPLC™ system (GE Healthcare) at 4°C. The HisTrap™ HP affinity column had been equilibrated previously with IMAC binding buffer A (50 mM Tris/HCl, pH 7.5, 250 mM NaCl and 25 mM imidazole). After loading the cell lysate, the HisTrap™ HP affinity column was washed with IMAC binding buffer A until a constant *A*_280_ was observed. His_6_-tagged TssK was eluted using a linear concentration gradient of 0–500 mM imidazole in IMAC elution buffer B (50 mM Tris/HCl, pH 7.5, 250 mM NaCl and 500 mM imidazole) over 12 column volumes. The eluate was collected in 1 ml fractions and analysed by Coomassie Blue-stained SDS/PAGE, and TssK-containing fractions were pooled and stored at 4°C. To remove the His_6_ tag, the pooled fractions were incubated with His_6_-tagged TEV protease at room temperature while dialysing against IMAC binding buffer A for 1 h then incubated at 30°C for 3 h. The sample was reapplied to the HisTrap™ HP affinity column and eluted as described above to separate the untagged protein (present in the flowthrough) from any residual His_6_-tagged recombinant protein, His_6_ tag and the His_6_-tagged TEV protease. Fractions were analysed by SDS/PAGE (15% gel) and those containing the untagged recombinant protein were pooled and stored at 4°C. The protein was purified further by SEC (size-exclusion chromatography) using a pre-packed Superdex 200 26/600 (GE Healthcare) previously equilibrated with gel filtration buffer (50 mM Tris/HCl, pH 7.5, and 250 mM NaCl). The pooled protein fractions were concentrated to 0.5 ml using an appropriate molecular-mass cut-off spin concentrator (Millipore) and applied to the SEC column using a 1 ml sample loop. Fractions of 0.5 ml were collected at a flow rate of 0.5 ml/min, and the level of sample purity in each fraction was determined by SDS/PAGE (15% gel). Columns were calibrated previously with high- and low-molecular-mass standards: Blue Dextran (>2000 kDa), thyroglobulin (669 kDa), ferritin (440 kDa), aldolase (158 kDa), conalbumin (75 kDa), ovalbumin (43 kDa), carbonic anhydrase (29.5 kDa), RNase A (13.7 kDa) and aprotinin (6.5 kDa) (GE Healthcare).

For co-purification of TssK–TssF–TssG, His_6_–TssF, TssG and TssK were co-produced in *E. coli* BL21(DE3) cells from pSC168 and the complex was purified using protocols described previously [19] and similar to above. In brief, following overproduction, the complex was isolated by an initial IMAC step, then the His_6_ tag on TssF was cleaved, followed by a negative IMAC step and finally two rounds of SEC using a calibrated Superose 6 10/300 GL column (GE Healthcare). Quantitative in-gel SYPRO Orange staining was performed as described in [47], with image analysis using ImageJ (NIH). Molar ratios resulted from the mean of at least four quantifications.

### Negative-staining electron microscopy

Immediately after gel filtration, purified TssK was diluted to 0.1 mg/ml in 50 mM Tris/HCl (pH 7.5) and 250 mM NaCl, collected on carbon film on mica and stained with 0.05 M uranyl acetate in 0.05 M HCl. The film was collected on an uncoated mesh copper grid, blotted dry and viewed in a Jeol 1200EX transmission electron microscope. Images were captured on Ditabis Micron imaging plates (Digital Biomedical Imaging Systems), scanned at 6000×5000 0.15 μm pixels and assembled into Figures using Adobe Photoshop.

### Analytical ultracentrifugation

The oligomeric state of pooled fractions of TssK from within Peak 1 and Peak 2 was ascertained via AUC (analytical ultracentrifugation) experiments performed at 4°C using an Optima XL-I analytical ultracentrifuge (Beckman Coulter) and an An-50 Ti eight-hole rotor. Samples were loaded into 12 mm pathlength charcoal-filled epon double-sector centrepieces, with 50 mM Tris/HCl (pH 7.5) and 250 mM NaCl buffer as reference solvent. SV (sedimentation velocity) was measured for samples (360 μl) at concentrations from 0.2 to 0.7 mg/ml (for Peak 1) and 0.2 and 0.5 mg/ml (for Peak 2). A series of sedimentation traces was collected every 7 min at a rotor speed of 49000 rev./min using interference optics and absorbance optics. Data were recorded over the radial range 6.0–7.25 cm, and a radial step size of 0.005 cm was used in the acquisition of absorbance data. In order to optimize the quality of the interference data, the laser delay and other related settings were adjusted before data acquisition. Data were analysed using the program SEDFIT [48]. Of the 64 sedimentation traces acquired, only 35 were used in the analysis, since the sample had reached the cell base by trace 35. Sedimentation boundaries were modelled as numerical finite element solutions of the Lamm equation using *c*(*s*) analysis. Apparent weight average sedimentation coefficients for Peak 1 were obtained by integrating over the peaks in the *c*(*s*) distributions. The partial specific volume ν of TssK calculated from its amino acid composition using the program SEDNTERP [49] was 0.737 ml/g at 20°C and 0.730 ml/g at 4°C. SEDNTERP was also used to calculate the density and viscosity of the buffer at 20°C and 4°C (1.00994 and 1.01173 g/ml, and 0.01040 and 0.01627 P respectively). As both SV absorbance and interference data yielded the same results, only absorbance data have been presented. SE (sedimentation equilibrium) was undertaken for samples (90 μl) at concentrations from 0.2 to 1.1 mg/ml. The distribution of TssK was monitored by interference optics at 3-h intervals at a rotor speed of 9000 rev./min until the system had reached equilibrium. This was ascertained using the program WinMATCH (Jeffrey Lary, University of Connecticut, Storrs, CT, U.S.A.) and equilibrium distributions at each loading concentration were analysed with the program SEDPHAT [50].

### Affinity purification of chromosomal His_6_-tagged TssK protein

*S. marcescens* strains were grown at 30°C for 5 h in 500 ml of LB medium. Cells were harvested by centrifugation at 3500 ***g*** for 45 min at 4°C, washed once with 10 ml of 50 mM Tris/HCl (pH 7.5), then snap-frozen in liquid nitrogen. The thawed cell pellet was resuspended in resuspension buffer (50 mM Tris/HCl, pH 7.5, 250 mM NaCl, 25 mM imidazole, 1:200 dilution of EDTA-free protease inhibitor cocktail set III and a few crystals of DNase I and lysozyme; 5 ml/g of cell pellet) and the cells were lysed with four passes through an Emulsiflex French press-style high pressure cell disruptor (20000 psi; Avestin). Cell debris was removed by centrifugation at 14100 ***g*** for 30 min at 4°C and the supernatant was retained for further ultracentrifugation at 100000 ***g*** for 1.5 h at 4°C. The supernatant was discarded and the remaining pellet, containing inner and outer membranes, was resuspended in 0.5 ml of 20% (w/v) sucrose (in 50 mM Tris/HCl buffer containing a 1:200 dilution of EDTA-free protease inhibitor cocktail set III) and gently homogenized on ice to avoid foaming. The homogenized total membrane fraction was then applied to a discontinuous sucrose gradient to separate inner and outer membranes, as above. Inner membrane-containing fractions were pooled and Triton X-100 was added dropwise to a final concentration of 2% (v/v), then samples were incubated for 2 h at 4°C and kept on ice until required. To prepare the magnetic Ni-NTA (Ni^2+^-nitrilotriacetate) beads (Qiagen) for affinity purification, 60 μl of magnetic beads were washed twice with 1 ml of wash buffer (20 mM Tris/HCl, pH 7.5, 100 mM NaCl, 25 mM imidazole and 0.1% Triton X-100). The inner membrane sample was added to the pre-washed magnetic beads and incubated for 2 h at 4°C with gentle end-over-end mixing to allow the binding of the TssK–His_6_ protein (and any interacting binding partners) to the Ni^2+^-charged beads. The magnetic beads were then washed thoroughly six times with 1 ml of wash buffer and the bound proteins were eluted by addition of 20 μl of 2× gel sample buffer and boiling for 2 min. Eluted proteins were resolved and analysed by SDS/PAGE (15% gel) and colloidal Coomassie Blue staining. Protein bands of interest were excised from the SDS/PAGE gel and subjected to in-gel trypsin digestion followed by one-dimensional nano-LC–ESI–MS/MS using a 4000 QTrap (Applied Biosystems) tandem-MS system by the FingerPrints Proteomics Facility, College of Life Sciences, University of Dundee. Raw QTrap data was analysed using MASCOT software for protein identification.

## RESULTS

### TssK is essential for T6SS activity in *S. marcescens*

**Figure 1:**
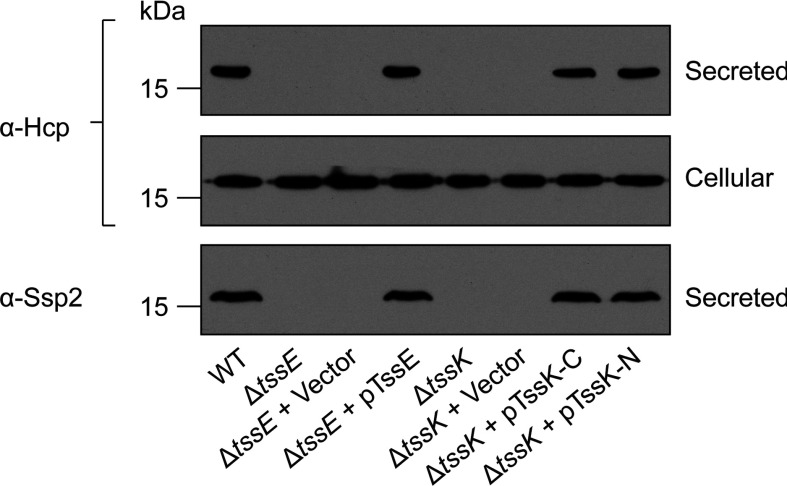
TssK is required for Type VI secretion by *S. marcescens* Anti-Hcp1 immunoblot of cellular and secreted proteins and anti-Ssp2 immunoblot of secreted proteins from wild-type *S. marcescens* Db10 (WT), T6SS mutants (SJC11, Δ*tssE*; GE04, Δ*tssK*) and T6SS mutants carrying either vector control plasmids (Δ*tssE* + Vector, pSUPROM; Δ*tssK* + Vector, pSC100) or complementing plasmids (Δ*tssE* + pTssE, pSC045; Δ*tssK* + pTssK-C, pSC521; Δ*tssK* + pTssK-N, pSC522). The volume of sample loaded in each lane for a given antibody corresponds to the same number of cells. Molecular masses are indicated in kDa.

A gene (*SMA2253*) encoding a 488-amino-acid homologue of the core component TssK is located within the *S. marcescens* Db10 T6SS gene cluster [18]. In order to determine the requirement of TssK for the basic function of the T6SS in *S. marcescens*, an in-frame chromosomal deletion of *tssK* was constructed. The ability of this mutant to secrete Hcp1 and the effector protein Ssp2, known substrates of the system [18,19], to the culture supernatant was determined by immunoblotting. For controls, culture supernatant from the wild-type strain and an inactive T6SS mutant lacking an essential component, Δ*tssE*, was also probed. As shown in Figure 1, deletion of *tssK* abolished secretion of both Hcp1 and Ssp2. This secretion defect could be fully complemented by the expression of N-terminal and C-terminal His_6_-tagged versions of the missing gene *in trans*, confirming the essential contribution that this component makes to T6SS function in this organism.

### Native TssK localizes to the inner membrane despite not being an integral membrane protein

**Figure 2:**
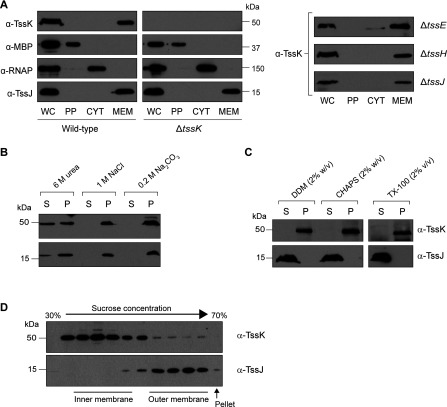
Subcellular localization of native TssK (**A**) TssK localizes to the total membrane fraction. Cells of wild-type *S. marcescens* Db10 (WT) and T6SS mutants (GE04, Δ*tssK*; SJC11, Δ*tssE*; SJC3, Δ*tssH*; SJC10, Δ*tssJ*) were subjected to fractionation as described in the Experimental section (WC, whole-cell extract; PP, periplasm; CYT, cytoplasm; MEM, membranes). Fractions were analysed by immunoblotting using antibodies against TssK, MBP (periplasmic marker), RNAPβ (cytoplasmic marker) and TssJ (membrane marker) as indicated. All fractionation controls are included for the wild-type and Δ*tssK* strains; for the other mutants, controls behaved identically with those shown. The volume of sample loaded in each lane for a given antibody corresponds to the same number of cells. The predicted mass of TssK is 50.4 kDa. (**B** and **C**) TssK association with the membrane fraction is resistant to salt, carbonate and detergent treatment. Total membrane pellets of wild-type *S. marcescens* Db10 were incubated with 50 mM Tris/HCl (pH 7.8) containing 6 M urea, 1 M NaCl or 0.2 M Na_2_CO_3_ (**B**), or with 50 mM Tris/HCl (pH 7.8) containing 2% (w/v) DDM, 2% (w/v) CHAPS or 2% (v/v) Triton X-100 detergents (**C**), then separated by ultracentrifugation into pellet (P) and soluble (S) fractions and analysed by immunoblotting using anti-TssK or anti-TssJ antibodies as indicated. (**D**) TssK is localized in the inner membrane. Inner and outer membranes of *S. marcescens* Db10 were separated by discontinuous sucrose gradient separation and fractions were analysed visually and by immunoblotting using antibodies against TssK and TssJ (outer membrane marker). Molecular masses are indicated in kDa.

In order to determine the subcellular localization of the native TssK protein in wild-type *S. marcescens*, an anti-TssK polyclonal antibody was utilized to probe TssK in each of the major cellular fractions. As shown in Figure 2(A), native TssK was found to localize exclusively in the membrane-containing fraction in the wild-type strain. This was unexpected as no part of the TssK protein is predicted to form classic hydrophobic transmembrane helices or amphipathic α-helical structures (results not shown). Thus, to examine the nature of the apparent membrane association of TssK, *S. marcescens* membranes were treated with various agents to discriminate between peripheral and integral membrane associations. The outer membrane lipoprotein TssJ [24] was used as a membrane-bound control. As shown in Figure 2(B), TssK and TssJ were resistant to extraction by salt or alkali, indicating that both proteins are not peripherally associated with the membranes. Partial solubilization of TssK by 6 M urea is consistent with presence of TssK in a stable membrane-bound complex (see below). Next, *S. marcescens* membranes were treated with the detergents DDM, CHAPS or Triton X-100 to solubilize conventional integral membrane proteins. Following treatment with the detergent and recentrifugation, TssJ was solubilized and found in the supernatant, whereas TssK remained in the pellet (Figure 2C). This result indicated that native TssK could be part of a membrane-bound complex that is resistant to detergent challenge or present in a complex or aggregate that is large enough to be pelleted by ultracentrifugation. Therefore, to determine further whether TssK was associated with the inner membranes, the outer membranes or a particulate fraction, discontinuous sucrose gradient centrifugation was employed to separate inner membranes, outer membranes and any particulate material (pellet). Using this method, bacterial inner membranes migrate to a position within the upper part of the gradient and outer membranes migrate within the lower part, in each case producing a distinct visible band [24,46]. Following separation, TssK was not detected at significant levels in the pellet or the outer membrane fraction (which was clearly defined both visually and using the outer membrane marker TssJ [24]). In contrast, TssK comigrated with the visually observed inner membrane band within the first half of the gradient (Figure 2D). Unfortunately, no antisera able to detect a specific inner membrane protein in this organism were available for direct comparison. Nevertheless, taken together, our data indicate that native TssK is present within an assembly localized to the inner membrane, presumably some or all of the T6SS apparatus.

Finally, we determined that the subcellular localization of the native TssK protein was unaffected in mutants carrying single in-frame deletions of three essential T6SS structural genes, *tssE*, *tssH* and *tssJ* (Figure 2A). TssJ is an outer membrane periplasm-facing lipoprotein that forms, together with TssL and TssM, the T6SS membrane complex [24,26], TssE may form part of a cytoplasmic baseplate-like structure [40] and TssH is the AAA+ protein required for remodelling TssBC complexes [38,39].

### Recombinant TssK can exist in different oligomeric states in solution

**Figure 3:**
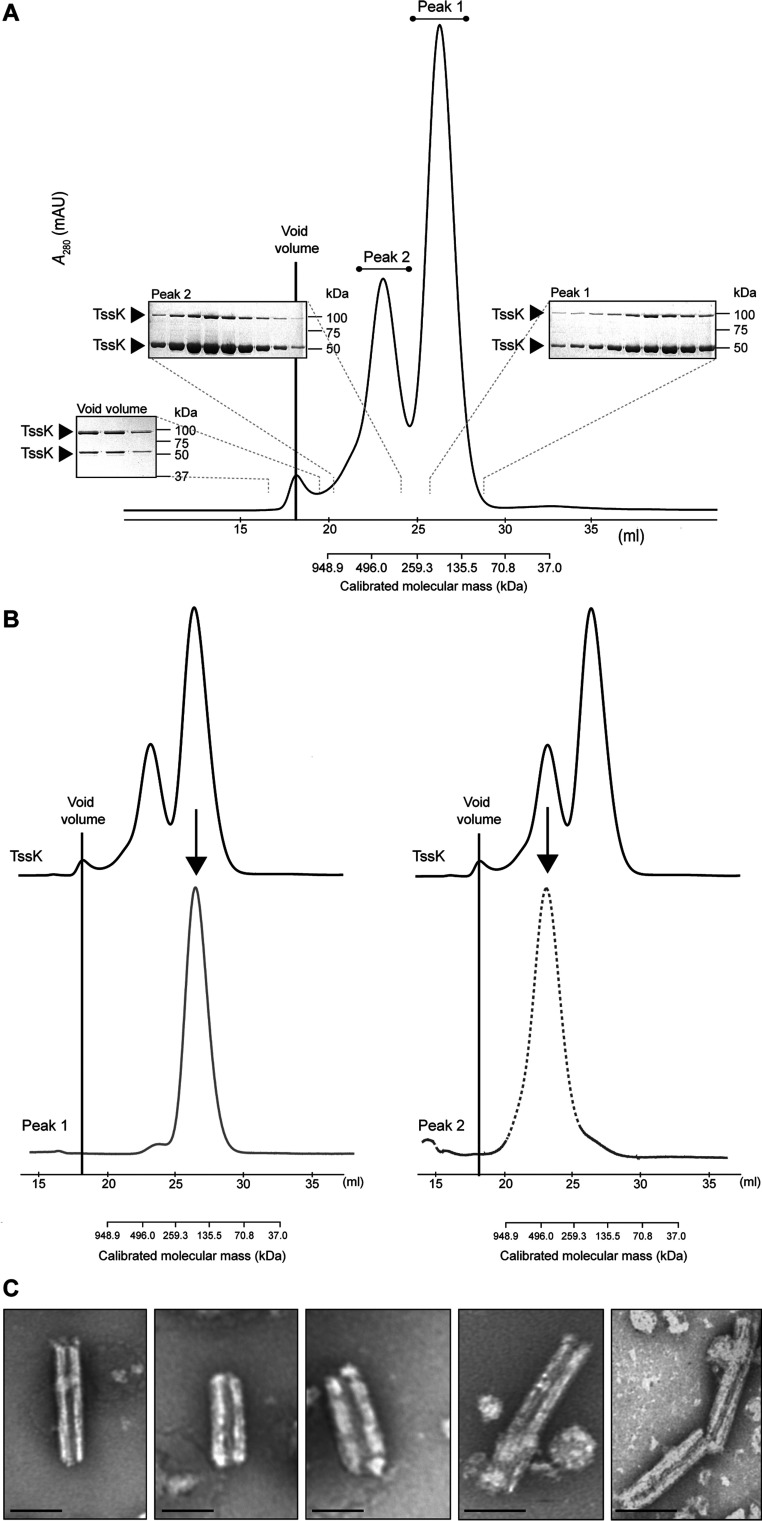
Recombinant TssK exists in different stable oligomeric states in solution (**A**) SEC analysis of recombinant TssK using a calibrated Superdex 200 26/60 column. Two peaks at elution volumes apparently consistent with tetramer (~200 kDa, Peak 1) and octamer (~400 kDa, Peak 2) forms were detected, in addition to a peak corresponding to the void volume of the column. Insets: analysis of peak fractions by SDS/PAGE and Coomassie Blue staining. The bands present at ~50 and ~100 kDa and identified as TssK by MS are indicated by arrowheads. (**B**) Further analysis of the fractions from Peak 1 and Peak 2 in (**A**). Fractions from each peak were pooled individually and subjected to a second round of SEC using the same column. The original trace is shown on top (black line) and the re-injected trace is shown below for Peak 1 (bottom left, continuous grey line) and Peak 2 (bottom right, broken grey line). The void volume is indicated. (**C**) Representative electron micrographs of the negatively stained TssK from the void volume fraction from (**A**). Scale bar, 50 nm.

With the aim of performing biochemical analysis of the protein, recombinant TssK was overexpressed and purified to homogeneity from the native organism, wild-type *S. marcescens* (Figure 3A). When subjected to SEC, two distinct peaks of size and composition were separated. These were suggestive of tetra-meric (~200 kDa; ‘Peak 1’) and octameric assemblies of TssK (~400 kDa; ‘Peak 2’). An SDS-resistant higher-order form of TssK with an apparent mass of ~100 kDa was also observed on analysis by SDS/PAGE. We wanted to investigate how stable the Peak 1 and Peak 2 forms of this protein were. The fractions corresponding to Peak 1 were pooled and subjected to a second round of SEC. As shown in Figure 3(B), the Peak 1 state of TssK was maintained and there was negligible detectable re-equilibration to the Peak 2 form. This was repeated for Peak 2 and, similarly, detectable re-equilibration to the Peak 1 form was not observed. Additionally, a third peak was observed in the original SEC chromatogram corresponding to the void volume fraction (Figure 3A). Analysis of this fraction by negative-staining EM unexpectedly revealed the presence of prominent high-molecular-mass oligomeric structures with a consistent diameter and an average length of 100–200 nm (Figure 3C).

**Figure 4:**
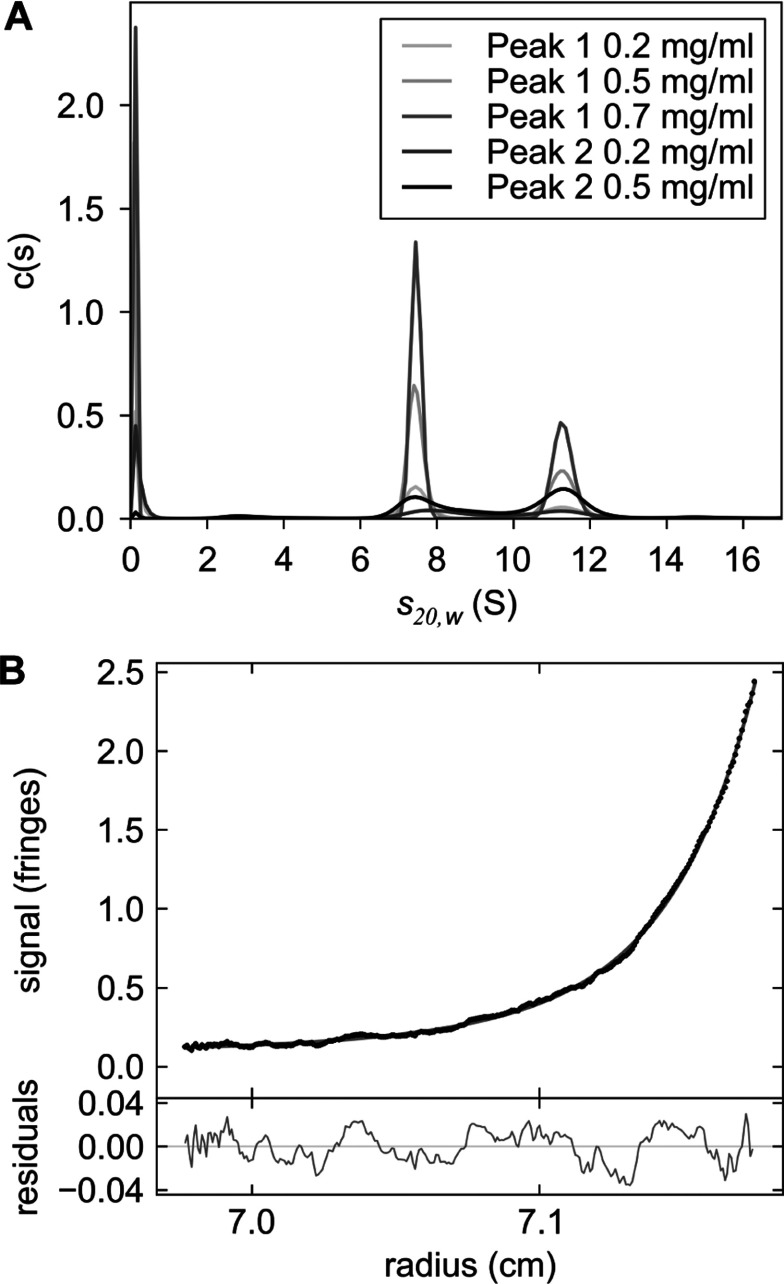
Analysis of the oligomeric states of recombinant TssK by AUC (**A**) Continuous distribution *c*(*s*) analysis of sedimentation velocity data for Peaks 1 and 2. The samples both contain two species with apparent sedimentation coefficients of 7.25 and 11.10 S. (**B**) Sedimentation equilibrium data for Peak 1 at a loading concentration of 0.2 mg/ml fitted with a three-species model in which the masses of two species were fixed at masses corresponding to trimeric and hexameric TssK and the third allowed to vary. Residuals of the fit are shown below. Plots were rendered with the program GUSSI (http://biophysics.swmed.edu/MBR/software.html).

In order to determine the oligomeric state of TssK in Peak 1 and Peak 2, fractions within each peak were pooled and analysed by AUC (Figure 4). Using SV, TssK from Peak 1 or Peak 2 was found to comprise two species with apparent sedimentation coefficients of 7.25 and 11.10 S at the concentrations analysed (Figure 4A). A spherical molecule of mass corresponding to that of a trimer of TssK with a plausible hydrodynamic hydration of 0.4 g of water/g of protein would have a sedimentation coefficient of 8.5 S, whereas that of a spherical tetramer would be 10.4 S. The non-spherical shape of a real protein complex will reduce the sedimentation coefficient from that of a sphere, but probably not by as much as 3 S since the SV data do not indicate the hydrodynamic non-ideality that would result from such a level of molecular asymmetry. So the experimentally determined value of 7.25 S is highly suggestive of a trimer, rather than a tetramer. The sedimentation coefficients computed for equivalent hydrated spheres for a hexamer and an octamer are 13.5 and 16.4 S respectively. Comparison with the experimental value (11.10 S) is indicative of a hexamer, as opposed to an octamer.

SE was performed at a rotor speed suitable for observation of species ranging in mass from a dimer to an octamer of TssK. With the knowledge that two species were observed in all samples by SV, the SE data were fitted to a two species model, with masses corresponding to a trimer and a hexamer of TssK. However, the fits were improved upon the addition of a third, higher-molecular-mass, species (Figure 4B). The mass of this species was not constant across the concentration range for Peaks 1 and 2 (results not shown) and was in excess of the upper limit properly observable at the rotor speed used (octamer). Also, the proportion of the sample modelled by the different species was significantly altered: for instance, the SV data for Peak 1 at 0.2 mg/ml were fitted with 60% ‘trimer’ species and 40% ‘hexamer’ species, whereas the SE data were fitted with 20% ‘trimer’, 30% ‘hexamer’ and 50% ‘big-mer’. This higher-molecular-mass species was absent from the SV data, although, if present, it would have been observable at the rotor speed and data acquisition time interval used. The SE data were acquired for the TssK samples that had been studied by SV 24 h previously. It is possible that we observed a very slow process of higher oligomer formation, reflected in the third peak of the original SEC chromatogram. Thus, in summary, the SE data are highly suggestive of a system that contains slowly declining populations of trimeric and hexameric species, at the cost of an evolving proportion of larger complexes.

### Isolation of a native TssK-containing complex: identification of TssF and TssG as interaction partners of TssK

**Figure 5:**
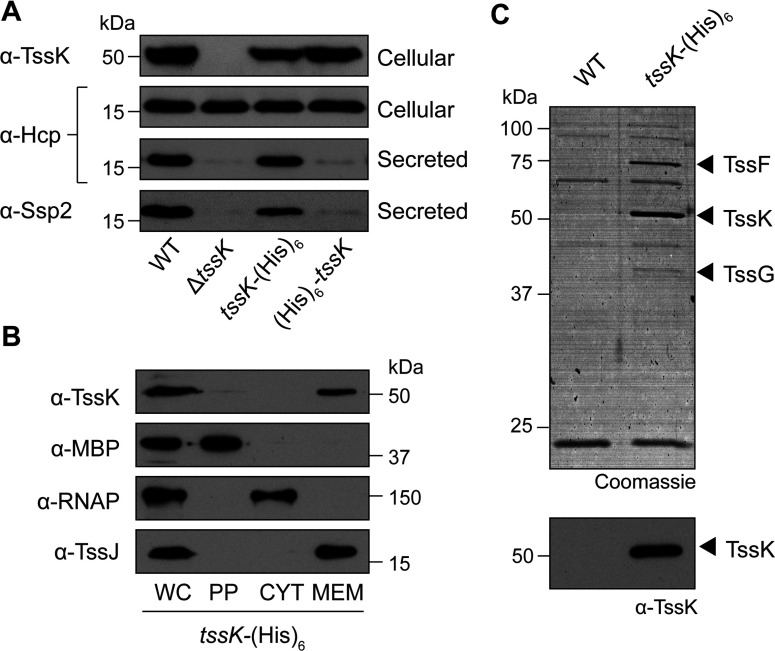
Isolation of a native TssK-containing complex (**A**) Anti-TssK, anti-Hcp1 and anti-Ssp2 immunoblots of cellular or secreted proteins, as indicated, from wild-type *S. marcescens* Db10 (WT), a strain carrying an in-frame deletion of *tssK* (GE04, Δ*tssK*) and strains encoding N-terminally His_6_-tagged TssK (SJC23; His_6_–*tssK*) and C-terminally His_6_-tagged TssK (MJF1; *tssK*–His_6_) at the native chromosomal location. (**B**) Subcellular localization of chromosomally encoded C-terminally His_6_-tagged TssK protein. The strain encoding His_6_-tagged TssK (MJF1; *tssK*–His_6_) was subjected to fractionation as described in the text (WC, whole-cell extract; PP, periplasm; CYT, cytoplasm; MEM, membranes). Fractions were analysed by immunoblotting using antibodies against TssK, MBP (periplasmic marker), RNAPβ (cytoplasmic marker) and TssJ (membrane marker) as indicated. (**C**) Identification of TssK-associated proteins by Ni^2+^-affinity purification (pull-down assay). Inner membrane fractions were prepared from the strain encoding chromosomal His_6_-tagged TssK (MJF1; *tssK*–His_6_), together with the wild-type (no His_6_ tag) as a negative control, and were incubated with magnetic Ni^2+^ beads to co-isolate TssK–His_6_ with any bound proteins. Top: eluted proteins were resolved by SDS/PAGE (15% gel) and visualized by colloidal Coomassie Blue staining. Black arrowheads indicate the positions of three bands specifically isolated from the TssK–His_6_ sample; these bands were identified as TssK, TssF (SMA2272, predicted mass 70.8 kDa) and TssG (SMA2273, predicted mass 41.1 kDa), as indicated, by MS. Bottom: anti-TssK immunoblot on the same pull-down samples. Molecular masses are indicated in kDa.

We hypothesized that TssK forms a complex with other T6SS components, perhaps contributing to the basal structure of the T6SS apparatus previously observed by microscopy [36]. We aimed to identify TssK-binding partners by isolating a native TssK-containing complex under physiological conditions, through affinity purification of TssK protein that was His_6_-tagged at the normal chromosomal location in *S. marcescens*. Strains bearing chromosomal His_6_ tag fusions to the N-terminus or C-terminus of the TssK ORF were constructed. Anti-TssK immunoblotting confirmed that TssK was expressed and stable in both strains, at levels comparable with that of the wild-type (Figure 5A). These strains were then tested to determine whether their T6SS was functional, by monitoring their ability to secrete Hcp1 and Ssp2. As shown in Figure 5(A), in the N-terminal His_6_–TssK fusion strain, the T6SS was non-functional, being unable to secrete Hcp1 or Ssp2. Thus modifying the N-terminus of TssK impairs its function sufficiently to abrogate T6SS function at native levels. However, the C-terminal fusion strain TssK–His_6_ was functional for T6SS activity, being able to secrete both Hcp and Ssp2 similarly to the wild-type strain. Consistent with its proper activity, the subcellular localization of TssK was also unaffected by the presence of the C-terminal His_6_ tag (Figure 5B). Thus this strain was used for downstream affinity purifications. Magnetic Ni^2+^-coupled matrix was used to affinity purify TssK–His_6_, together with any interacting protein partners, from inner membrane fractions prepared from the *S. marcescens* chromosomal *tssK*–His_6_ strain. Eluted proteins were separated by SDS/PAGE and stained with Coomassie Blue to visualize co-purified proteins. Protein bands present in the eluate of the TssK–His_6_ strain, but not the wild-type strain (negative control, encoding no His_6_-tagged proteins), were selected and subjected to MS identification (Figure 5C). Three TssK–His_6_-specific bands were identified as TssK itself (50 kDa) and as two other T6SS core proteins, TssF (SMA2272; 71 kDa) and TssG (SMA2273, 41 kDa). Hence, in this native pull-down experiment, TssF and TssG were isolated by virtue of their interaction with TssK.

**Figure 6:**
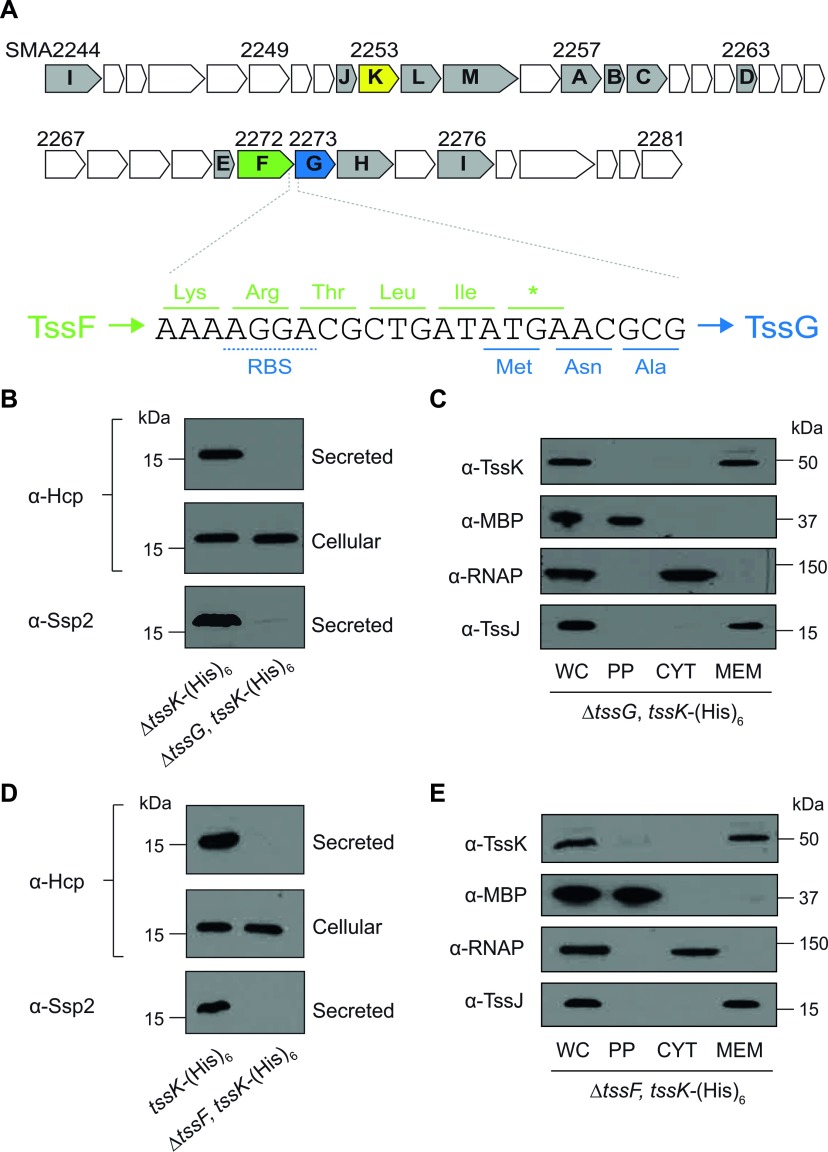
TssF and TssG are essential components of the T6SS, but are not required for TssK localization to the membrane (**A**) Schematic depiction of the genetic context of *tssF* and *tssG* illustrating their potential translational coupling. All of the genes in the *S. marcescens* Db10 T6SS gene cluster (*SMA2244*–*2281*) are shown, with core conserved components shaded and lettered according to the Tss nomenclature. TssF residues are shown in green; TssG residues are shown in blue. The position of the TssG ribosome-binding site (RBS) is underlined with a broken line; the TssF stop codon is shown as an asterisk. (**B** and **D**) TssG and TssF are required for Type VI secretion. Anti-Hcp1 immunoblot of cellular and secreted proteins and anti-Ssp2 immunoblot of secreted proteins from strains bearing in-frame deletions of *tssG* (GE05; Δ*tssG*,*tssK*–His_6_) (**B**) or *tssF* (GE06; Δ*tssF*,*tssK*–His_6_) (**D**), compared with the parental strain with wild-type T6SS activity (MJF1; *tssK*–His_6_). (**C** and **E**) TssK localization is not affected by deletion of TssG or TssF. Strains encoding His_6_-tagged TssK in Δ*tssG* background (GE05; Δ*tssG*,*tssK*–His_6_) (**C**) or Δ*tssF* background (GE06; Δ*tssF*,*tssK*–His_6_) (**E**) were subjected to fractionation as described in the text (WC, whole-cell extract; PP, periplasm; CYT, cytoplasm; MEM, membranes). Fractions were analysed by immunoblotting using antibodies against TssK, MBP (periplasmic marker), RNAPβ (cytoplasmic marker) and TssJ (membrane marker) as indicated. Molecular masses are indicated in kDa.

Having identified TssF and TssG as TssK-specific binding partners, we aimed to investigate further their role in Type VI secretion and in subcomplex assembly. Analysis of their genomic context revealed that TssF and TssG are encoded by overlapping genes in a manner consistent with mutual interaction of the proteins and likely translational coupling (Figure 6A). Indeed the genomic organization of TssF (COG3519) adjacent to TssG (COG3520) is highly conserved across T6SS gene clusters [51]. Strains carrying in-frame deletions of *tssF* and *tssG* were constructed in the chromosomal *tssK*–His_6_ background. The T6SS functionality of these new strains was tested and it was found that deletion of *tssF* or *tssG* completely abolished secretion of Hcp1 and Ssp2 (Figures 6B and 6D). This confirmed that TssF and TssG are essential components of the T6SS in *S. marcescens*. In contrast, Δ*tssF* and Δ*tssG* deletions did not affect the membrane localization of the native TssK protein (Figures 6C and 6E). Affinity purifications of TssK–His_6_ from Δ*tssF* or Δ*tssG* mutants failed to yield TssG or TssF respectively, suggesting that a pre-formed TssFG complex may interact with TssK (results not shown).

### TssK, TssF and TssG form a large stable complex *in vitro*

**Figure 7:**
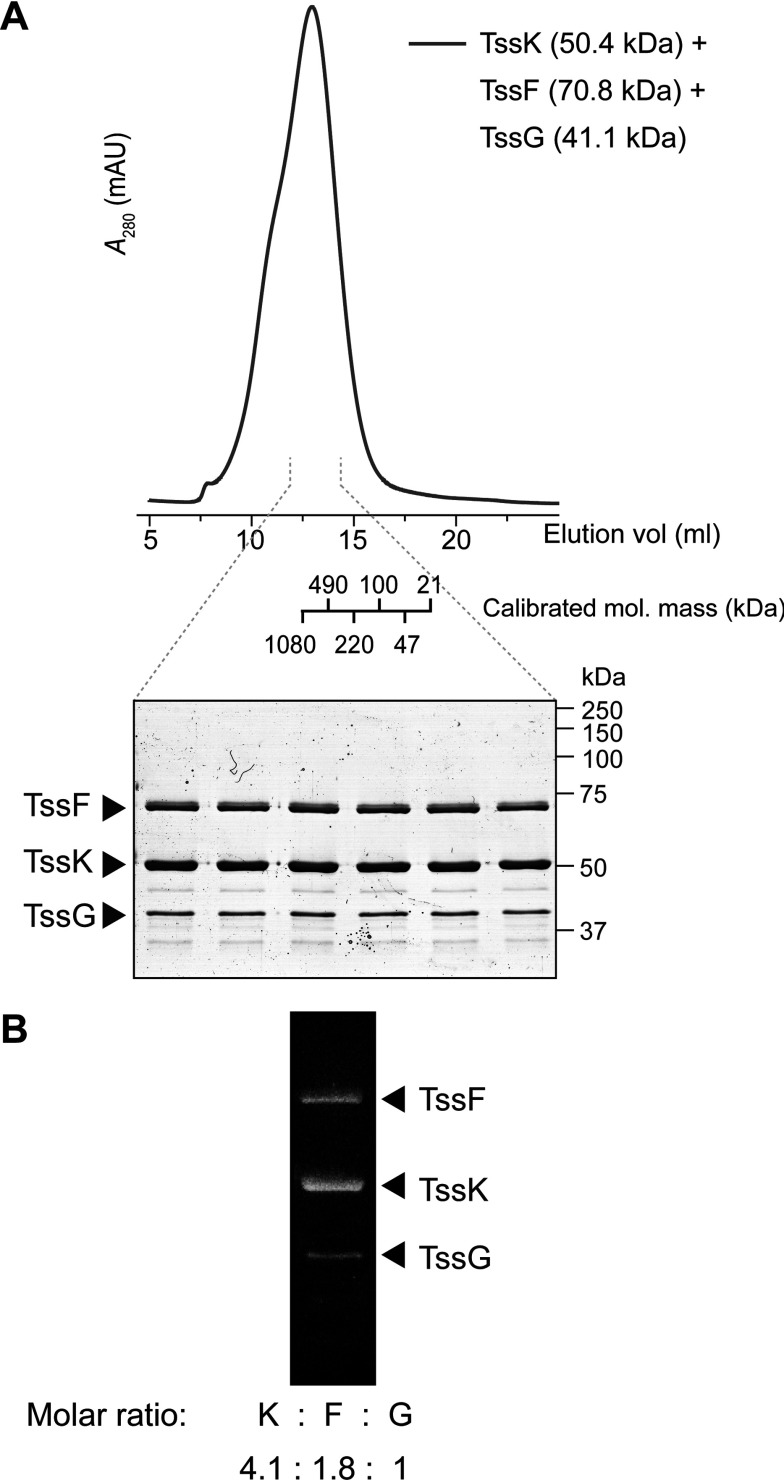
TssK, TssF and TssG form a stable complex *in vitro* (**A**) SEC profile of the complex formed between TssK, TssF and TssG. Separation was performed using a calibrated Superose 6 10/300 GL column. The proteins had previously been co-expressed in *E. coli* and the complex was initially purified by IMAC and negative IMAC, using a TEV protease-cleavable N-terminal His_6_ tag on TssF. The theoretical mass of each protein is given (kDa) and the complex has an apparent molecular mass of >670 kDa. (**B**) Analysis of the complex-containing peak observed in SEC by quantitative in-gel SYPRO Orange staining. Quantification of bound fluorescence gives a molar ratio of approximately 4:2:1 TssK/TssF/TssG.

To confirm the interaction of TssK with TssF and TssG identified *in vivo*, we purified the corresponding complex *in vitro*. The three proteins were co-expressed in *E. coli* and purified using a cleavable N-terminal His_6_ tag on TssF. Following purification by Ni^2+^-based IMAC, cleavage of the His_6_ tag and reverse IMAC, a complex containing the three proteins was analysed further by SEC (Figure 7A). The identity of each band was confirmed by MS and when the IMAC was repeated using a construct lacking His_6_–TssF, TssG and TssK were not isolated (results not shown). Hence this analysis confirmed the existence of a stable complex of TssF, TssG and TssK and indicated further that the complex contains multiple subunits, displaying an apparent molecular mass of >670 kDa (i.e. greater than that of the largest standard protein analysed on the column). To estimate the stoichiometry of each protein within the complex, we performed quantitative in-gel staining [47]. This gave a molar ratio of 4:2:1 TssK/TssF/TssG (Figure 7B). Given that the apparent molecular mass of the complex is far in excess of 385 kDa (4×TssK+2×TssF+TssG), we suggest that the stoichiometry of the complex could be 12:6:3 TssK/TssF/TssG, accommodating four trimers of TssK. Overall, it is clear that the TssFGK complex represents a physiologically relevant, stable and multisubunit subcomplex of the T6SS.

## DISCUSSION

In the present paper, we report the functional and biochemical characterization of one of the highly conserved proteins of the T6SS: TssK. We have shown that TssK is required for secretion of Hcp1 and Ssp2 and thus makes an essential contribution to the function of the T6SS machinery in *S. marcescens*. In agreement with this, TssK was found to be essential for the formation of visible foci of TssB–sfGPF in EAEC, suggesting that assembly of the T6SS could not proceed to incorporate the TssBC-containing contractile sheath [43]. Our subcellular localization of the native TssK protein found it to be associated with the membrane fraction, specifically the inner membrane. This is in agreement with the finding that TssK is present in the inner membrane fraction of wild-type *P. aeruginosa* [52]. Our analysis indicates that TssK is not an integral membrane protein, supported further by the detection of TssK in the cytoplasmic fraction when overexpressed in EAEC [43]. Rather, our data suggest that it is associated with the inner membrane by virtue of interactions with other T6SS proteins, within a membrane-associated assembly representing some or all of the T6SS apparatus. This complex is clearly highly stable, since TssK association with the membrane fraction cannot be abrogated by high salt, carbonate or detergent. We speculate that detergent resistance could be either as a result of the transmembrane protein domains of TssLM being shielded from the detergent by other proteins in the complex, or because the assembly is so large that it continues to sediment on ultracentrifugation even when released from the lipid bilayer.

During initial structural and biochemical analyses of TssK, it became apparent that recombinant TssK protein, purified from the native T6SS-harbouring *S. marcescens*, exhibited striking oligomerization properties. Stable trimeric, hexameric and higher-order species were observed in solution. These results demonstrate that TssK has a propensity to self-interact and suggest that it has the ability to form large structures. The biological significance of these large structures and the form, if any, that they take at native levels *in vivo* are yet to be determined (see below). Our attempts to crystallize TssK were unsuccessful. Its existence in multiple oligomeric forms in solution (both when purified from *S. marcescens* or *E. coli*; results not shown) probably explains this lack of success. The recent study by Zoued et al. [43] is in accord with our observation of a basic trimeric unit for TssK. The authors observed that TssK from EAEC exists as a trimer in solution, with EM and SAXS analyses suggesting that it displays a ‘three-armed’ structure [43]. However, our work on TssK from *S. marcescens* has demonstrated for the first time that TssK can adopt an alternative, stable, hexameric state in solution and has the potential to form higher-order oligomers, at least *in vitro*.

**Figure 8:**
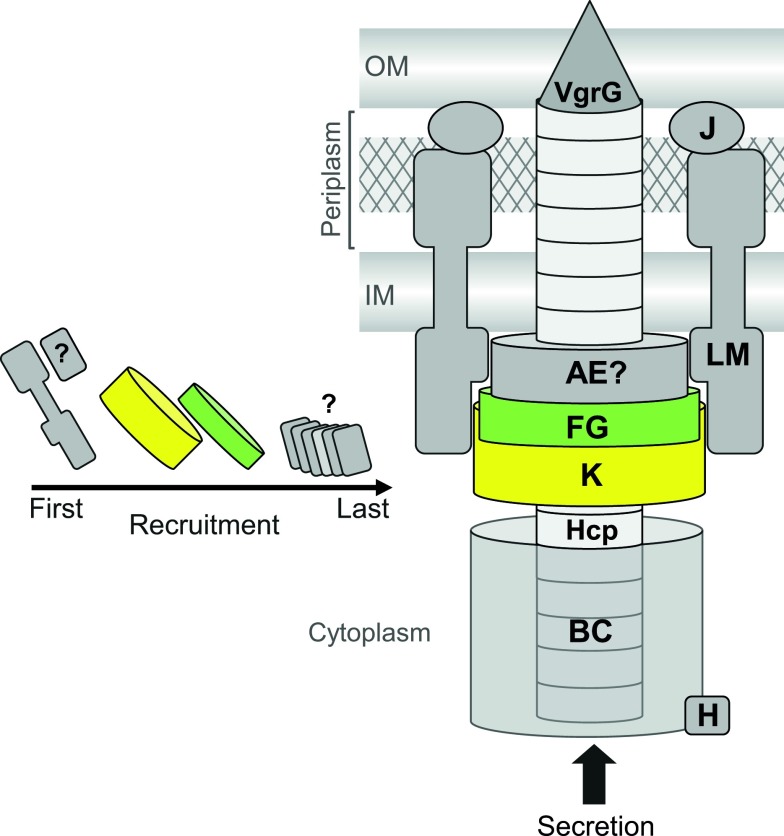
Speculative model of the assembly of TssK, TssF and TssG into the T6SS The inner membrane components of the T6SS, TssL and TssM, are in place first, marking the site of T6SS assembly and anchoring the complex to the inner membrane. TssK is subsequently recruited to the assembly and becomes tethered to the membrane by interacting with TssLM, either directly or through interactions with other basal components recruited ahead of it, for example TssA. TssFG units are next recruited to the basal complex through their interaction with TssK. Subsequently, other components of the machinery, including the Hcp tube, TssBC sheath and TssH, are recruited. TssJ can join TssLM to complete the membrane subassembly at any point, but this is not required for TssK recruitment. In the final T6SS assembly, TssKFG form a subcomplex within the basal complex anchored to the cytoplasmic side of the inner membrane which probably also contains TssA and TssE. This basal structure is connected to the membrane complex, Hcp tube and the TssBC sheath, allowing contraction of the sheath to propel Hcp, VgrG and secreted proteins out of the cell. TssK itself may be involved in mediating the interaction of the Hcp tube with the basal complex and/or may contribute to the dynamic mechanism of the system through a conformational change within the TssKFG subcomplex. Multimeric TssK is shown in yellow and the TssFG complex in green; letters A–M represent TssA–TssM; IM, inner membrane; OM, outer membrane.

By utilizing a chromosomal epitope-tagging approach, where the protein is expressed at normal levels in concert with all of the other proteins of the system, a native TssK-containing complex was isolated. This revealed, for the first time, that TssK interacts with two other core conserved components, TssF and TssG, forming a newly identified T6SS subcomplex. The interaction of TssK–TssF–TssG was confirmed by the isolation of a complex containing the three proteins *in vitro*. The maintenance and detection of the interaction through a native-level affinity purification, together with the isolation of a stable complex *in vitro*, strongly suggests that the interactions are relatively strong and a TssKFG complex is a physiologically relevant composite part of the T6SS. The molar ratio of the three proteins is 4:2:1 (TssK/TssF/TssG), which, together with the large size of the complex, allows us to suggest a possible stoichiometry of 12:6:3 (TssK/TssF/TssG). However, although it would be clearly predicted that these proteins will be in multiple copies, the stoichiometry of TssK, TssF and TssG *in vivo*, within the entire T6SS assembly, remains to be determined. TssF and TssG were confirmed to represent essential components of the T6SS in *S. marcescens*, in agreement with systematic mutagenesis studies in *E. tarda* and *V. cholerae* showing that each is required for T6SS function [41,42]. Our data also suggest that they interact with each other, probably before interaction with TssK. Importantly, TssF and TssG are not required for the recruitment of TssK to the membrane fraction. This suggests that, during T6SS assembly, a complex of TssLM, the only integral inner membrane proteins within the T6SS, is in place first, anchoring the nascent machinery to the inner membrane, with TssK and then TssFG recruited subsequently (depicted in our proposed model, Figure 8). In support of this idea, TssK has been reported to interact with TssL [43]. Interestingly, none of TssJ, TssE or TssH is required for TssK localization, therefore they may be recruited to the machinery in the later stages of assembly. Indeed, there is already evidence that TssH is recruited very late, since it associates with contracted TssBC sheaths [39,53]. Hence all three proteins, i.e. TssK, TssF and TssG, play an important role in the assembly of the T6SS machine and form another T6SS subcomplex, in addition to, for example, the TssJLM or TssBC subcomplexes.

The present study is the first to identify a TssK-containing complex under native conditions, demonstrating that TssK stably interacts with TssFG. The recent study in EAEC suggested that TssK can interact with Hcp and TssC (tail tube and sheath), TssL (membrane complex) and TssA (putative cytoplasmic/basal component), by detection of bacterial two-hybrid interaction or co-immunoprecipitation between pairs of proteins expressed heterologously [43]. The TssK–Hcp interaction, with a *K*_d_ of 27 μM, was confirmed *in vitro* and suggests that TssK may be involved in connecting the phage tail-like structure of the T6SS with a basal complex. However, an interaction between TssK and TssF or TssG was not detected using this plasmid-based approach. Our data suggest that this is because TssK only interacts with a TssFG complex and not with the individual proteins, either because the complex itself is recognized and/or because TssF and TssG are unstable in the absence of the other. Indeed, when we attempted to purify TssF and TssG separately for binding studies between these proteins and TssK, we were unable to overproduce TssG or to isolate soluble TssF (results not shown), suggesting a mutual stability requirement and consistent with putative translational coupling between the proteins (Figure 6A). Our successful identification of the TssKFG complex illustrates the value of identifying interactions at native protein levels, in the native organism, where all components of the system are present and in the correct stoichiometry. The fact we did not observe TssL, TssC or Hcp in our native pull-downs may indicate that the interactions of these proteins with TssK are too weak or transient to survive the purification at detectable levels.

It has been observed by microscopy that the base of the contractile TssB–TssC sheath-like structure is connected to the inner membrane by a large cytoplasmic bell-shaped ‘basal’ structure that is yet to be fully characterized [36]. It is thought that this large complex may represent an assembly platform similar to the bacteriophage baseplate, a structure responsible for contraction of the bacteriophage tail sheath and correct assembly of the tail hub and tube [23,54]. It has been speculated that the core T6SS components, i.e. TssA, TssE, TssF, TssG and TssK, may form part of the T6SS cytoplasmic basal structure [55]. Indeed, TssE has been shown to display significant sequence homology with bacteriophage T4 gp25, a structural component of the baseplate, and is localized in the cytoplasm [34,40]. It is thought that TssE, with other components of the T6SS basal complex, might contribute to the assembly of the Hcp tube and the TssB–TssC sheath structure [23]. However, no obvious similarities or homologies were detected between TssF, TssG or TssK and bacteriophage components at the sequence level [22]. The T6SS basal structure probably also includes, or interacts with, the cytoplasmic domains of the inner membrane proteins within the membrane complex, anchoring the machine to the inner membrane. Consistent with TssK being part of such a basal complex, native-level purification of TssK revealed for the first time an interaction between this protein and two other core components also predicted to reside in a cytoplasmic T6SS basal complex, TssF and TssG. In bacteriophage T4, the baseplate is composed of 16 different proteins and has six-fold symmetry with a diameter of 52 nm around its base and a height of 27 nm [54]. However, how similar a T6SS basal complex containing TssKFG, and perhaps also TssA and/or TssE, actually is to the phage baseplate remains to be determined.

The *in vivo* significance of the large EM-visible structures formed by purified TssK, if any, is currently unclear. However, the stability of the trimer and hexamer forms of recombinant TssK indicates these forms are likely to exist *in vivo*. We propose that post-translational modification of TssK or its interaction with other T6SS proteins may trigger a ‘switch’ between these different forms which may in turn mediate conformational changes involved in the dynamic steps of Type VI secretion. Of note, a minor ‘shoulder’ on the SEC profile of the TssKFG complex (Figure 7B) may indicate the existence of a second form of this TssK-containing complex. The ability of recombinant TssK alone to further form tubular-like structures *in vitro* may indicate a propensity to associate into ring-like structures. Thus, if TssK does form part of the T6SS baseplate, it is likely to be present in multiple copies and perhaps form rings. ‘Excessive’ polymerization of TssK may be limited *in vivo* by its interaction with other components of the dynamic T6SS apparatus, perhaps TssFG.

In conclusion, we have shown for the first time that the core T6SS component TssK can exist in several stable oligomeric states, provided evidence that it localizes at the inner membrane in a manner consistent with its presence in a membrane-anchored T6SS basal structure, and revealed that it interacts with TssF and TssG complex at native levels to form a new subcomplex of the system. Furthermore, our results and those of others, described above, have allowed us to propose a model for the assembly of TssK, TssF and TssG into the T6SS machinery (Figure 8). It is clear that TssK represents a central component of the T6SS machine. Thus it may represent a future target for the development of antimicrobial therapies aimed at disrupting the T6SS and hence disabling the many pathogenic bacteria which use T6SSs to attack competitor bacteria or host cells.
